# Enhancing Psychosocial Support for HIV Positive Adolescents in Harare, Zimbabwe

**DOI:** 10.1371/journal.pone.0070254

**Published:** 2013-07-23

**Authors:** Webster Mavhu, Jessica Berwick, Petronella Chirawu, Memory Makamba, Andrew Copas, Jeffrey Dirawo, Nicola Willis, Ricardo Araya, Melanie A. Abas, Elizabeth L. Corbett, Stanley Mungofa, Susan M. Laver, Frances M. Cowan

**Affiliations:** 1 Zimbabwe AIDS Prevention Project, Department of Community Medicine, University of Zimbabwe College of Health Sciences, Harare, Zimbabwe; 2 Centre for Sexual Health and HIV/AIDS Research Zimbabwe (CeSHHAR Zimbabwe), Harare, Zimbabwe; 3 Centre for Sexual Health and HIV Research, Research Department of Infection and Population Health, University College London, London, United Kingdom; 4 Johns Hopkins Bloomberg School of Public Health, Baltimore, Maryland, United States of America; 5 Africaid, Harare, Zimbabwe; 6 School of Social and Community Medicine, Bristol, United Kingdom; 7 Institute of Psychiatry, King’s College, London, United Kingdom; 8 London School of Hygiene and Tropical Medicine, London, United Kingdom; 9 Harare City Health Department, Harare, Zimbabwe; 10 CCORE (Collaborating Centre for Operational Research & Evaluation), UNICEF, Harare, Zimbabwe; University of Ottawa, Canada

## Abstract

**Background:**

There is a recognized gap in the evidence base relating to the nature and components of interventions to address the psycho-social needs of HIV positive young people. We used mixed methods research to strengthen a community support group intervention for HIV positive young people based in Harare, Zimbabwe.

**Methods:**

A quantitative questionnaire was administered to HIV positive Africaid support group attendees. Afterwards, qualitative data were collected from young people aged 15–18 through tape-recorded in-depth interviews (n = 10), 3 focus group discussions (FGDs) and 16 life history narratives. Data were also collected from caregivers, health care workers, and community members through FGDs (n = 6 groups) and in-depth interviews (n = 12). Quantitative data were processed and analysed using STATA 10. Qualitative data were analysed using thematic analysis.

**Results:**

229/310 young people completed the quantitative questionnaire (74% participation). Median age was 14 (range 6–18 years); 59% were female. Self-reported adherence to antiretrovirals was sub-optimal. Psychological well being was poor (median score on Shona Symptom Questionnaire 9/14); 63% were at risk of depression. Qualitative findings suggested that challenges faced by positive children include verbal abuse, stigma, and discrimination. While data showed that support group attendance is helpful, young people stressed that life outside the confines of the group was more challenging. Caregivers felt ill-equipped to support the children in their care. These data, combined with a previously validated conceptual framework for family-centred interventions, were used to guide the development of the existing programme of adolescent support groups into a more comprehensive evidence-based psychosocial support programme encompassing caregiver and household members.

**Conclusions:**

This study allowed us to describe the lived experiences of HIV positive young people and their caregivers in Zimbabwe. The findings contributed to the enhancement of Africaid’s existing programme of support to better promote psychological well being and ART adherence.

## Introduction

Much progress has been made in reducing mother-to-child transmission of HIV over the past decade, with six countries in Africa reducing the number of new infections by over 40% [1], [2]. Even so, in 2011 there were an estimated 330,000 (280,000–390,000) new infections among children, most of whom were perinatally infected [3], [4] and over 90% of whom were living in sub-Saharan Africa [5]. The number of 0–14 year olds living with HIV globally is estimated to be 3.3 million (3.1–3.8 million) [3], [5]. In 2011, it was estimated that in Zimbabwe, 2.8% (1.6–3.7) of those aged <15 years old were HIV positive (138,642 HIV-infected children); 13,711 were newly infected in 2010 [6], [7]. A study among hospitalized young people in Harare found that 46% were admitted as a result of both recent and long-term HIV-related complications [8]. Antiretroviral therapy (ART) has been available in the public sector since 2004. By the end of 2011, 37,590 children aged <15 years were receiving ART nationally (personal communication Ministry of Health and Child Welfare [MoHCW]).

Young people who have survived HIV since birth face several challenges. Their childhoods have been characterized by frequent illness, hospitalization and poor school attendance; they are frequently of small stature, have delayed puberty, intellectual impairment and skin disfiguration [9], [10]. Many have lost one or both parents to HIV [10]. They need specialised psychosocial assistance to help them adjust to their diagnosis and where necessary, integrate into a new household. These challenges have implications for designing interventions in order to address their needs. Of note, many older children and adolescents are diagnosed late in Africa despite most guardians suspecting their children of being HIV infected before diagnosis [11], [12].

The needs of adolescents living with HIV are more sensitive and varied than those of adults, as they must simultaneously deal with ‘adult’ issues such as disclosure, stigma and practising safe sex while also addressing issues traditionally associated with adolescence, such as body image, first sexual experience, peer pressure and forming personal identity [13], [14]. In addition, while HIV treatment and care programmes in general and highly active ART specifically, has been demonstrated to improve paediatric HIV outcomes in developed [15], [16], [17], [18] and low-resource settings [19], high levels of adherence (>90%) [20], [21] are required to decrease the risk of virological resistance [22], [23] and opportunistic infections [24]. There is evidence that ART adherence in young people is worse than in adults [25], [26], [27]. Young people on ART need support in order to ensure the high levels of adherence required to maximise the benefits of therapy.

There is a recognized gap in the evidence relating to the optimal content and components of interventions to address the needs of HIV positive young people in order to support their transition to adulthood and their engagement in treatment and care services [14]. At the time the study was undertaken, the only randomized trial of a mental health intervention to improve ART adherence among people (all adults) with depressions was by Steve Safran and colleagues and this intervention used CBT [28]. In addition, the only preventive mental health intervention recommended for adolescents by the World Health Organisation and evaluated in various RCTs was the Friends intervention (which also uses CBT as its basis) [29], [30].

In Zimbabwe, psycho-social support (PSS) for HIV positive children is run through a number of organisations, the majority of whom provide PSS to vulnerable children more generally rather than HIV positive children specifically. The intensity and nature of such provision varies widely [31]. The MOHCW has established guidelines for PSS for HIV positive children. It has recognised seven principles for programming [32] and has also developed performance indicators but has not as yet determined a ‘standard of care’ for this type of provision. Africaid (*Zvandiri* – ‘As I am’) is one of the few organisations in Zimbabwe specifically targeting the PSS of positive young people and provides community-based support addressing the psychological, social and treatment needs of the young people in its care (www.africaid.co.uk).

In partnership with Africaid, we used mixed methods research to collect data to strengthen the evidence base for psychosocial interventions to support HIV positive adolescents in southern Africa, with the specific goal of strengthening Africaid’s community intervention for HIV positive young people. We used research findings to come up with a description of internal and external life circumstances of HIV positive young people which we could combine with stakeholder input and evidence from the literature about the determinants of adolescent HIV adherence to strengthen and expand Africaid's programme of support.

## Methods

All HIV positive young people (aged 6–18) enrolled in Africaid support groups during the study period were invited to take part in a quantitative survey. In addition, qualitative data were collected from young people using in-depth interviews (IDIs), focus group discussions (FGDs) and life history narratives. Additionally, IDIs and FGDs were held with parents and caregivers of HIV positive children, health-care workers and community members with different roles. We then held a participatory workshop with young people living with HIV, their caregivers and key stakeholders to review these data and ensure their credibility [33]. The data were discussed alongside the relevant published literature and implications for enhancing the existing community based programme were explored. Stakeholders recommended re-focusing of the programme in order that the research findings were taken into account. A previously validated theoretical framework for adolescent behaviour change (in this case adherence to ART) which took account of the stakeholders’ interpretation of research findings was identified [34], [35] and used to guide refocusing of the programme.

We used youth researchers to collect data from the HIV positive young people in an attempt to maximise participation and disclosure of sensitive issues. Involving youth researchers in research design, implementation and analysis as well as subsequent intervention development provided a framework for increasing child participation in research and programming. Between July and August 2009, 10 youth researchers aged 16–18 (6 females), carefully selected among Africaid support group attendees, were trained in data collection and analysis using materials adapted from the Save the Children framework for training youth researchers [36], [37]. The training covered research and ethical issues, data collection and recording, as well as questionnaire delivery.

Young people were recruited through Africaid support groups. At the time of the study, there were 310 support group attendees. Parents and caregivers who took part in IDIs and FGDs were recruited through the peer researchers. Health-care workers were recruited at their workplaces. Community members were approached within the community and purposively sampled to include individuals likely to have contact with HIV positive young people including teachers, the police and child protection committee members.

### Data Collection

**Figure 1:**
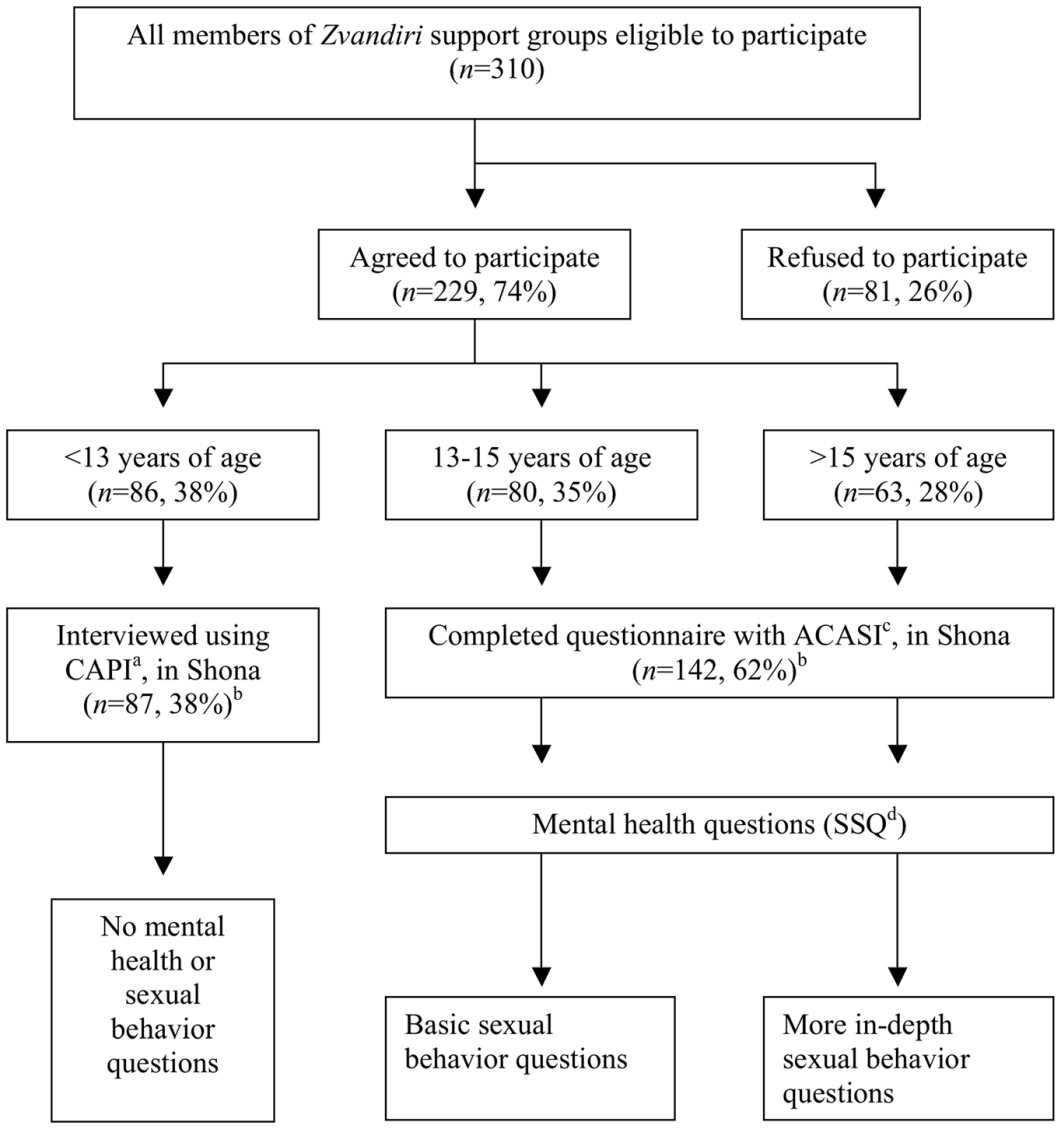
Flow chart of study participants, questionnaire mode and questions by age group. ^a^Computer assisted personal interview. ^b^Four 12 year old participants were mistakenly administered ACASI, and five participants <12 years of age were mistakenly administered CAPI. ^c^Audio computer assisted survey instrument. dShona Symptom Questionnaire.

Quantitative data were collected in Shona, the participants’ language, between August and September 2009 using a structured questionnaire. Two different questionnaire delivery modes were ascribed to different age groups. Young people below the age of 13 were interviewed by peer researchers using computer-assisted personal interview (CAPI) (Figure 1); participants’ responses were entered directly into Personal Digital Assistants (PDAs). The survey instrument was developed from previously validated questionnaires including those used for the Regai Dzive Shiri project - a randomized trial of an HIV prevention intervention for Zimbabwean youth [38], [39], the Zimbabwe Demographic and Health Survey [40] and the Zimbabwe Young Adult Survey [41]. Where existing questions were not available, these were developed de novo and pretested. Questionnaire domains included socio-demographics, disclosure, stigma and adherence. Questions relating to sexual behaviour and mental health symptoms were not presented to this age group. Young people aged between 13 and 18 self-completed the questionnaire on a laptop using audio computer-assisted survey instrument (ACASI). All participants aged 13 and older completed questions regarding their mental health. Participants aged between 13 and 15 were asked a few sexual behaviour questions; those over 15 years were asked about their sexual behaviour in more depth, including age at first sex, condom use and life-time sex partners.

After the quantitative survey, qualitative data were collected from young people aged 15–18 through tape-recorded, IDIs (n = 10), 3 FGDs (n = 30 participants) and a predetermined number of life history narratives/‘rivers of life’ (RoLs) (n = 16). A ‘river of life’ is a participatory tool whereby each participant chronicles their life through drawing a river, marking key events with either an upward (positive occurrence) or downward flow (negative event). The drawing serves as stimulus for an in-depth discussion of their life [42]. Youth researchers conducted some of the in-depth interviews and life history narratives on their own but received support from experienced adult researchers around FGD facilitation, which they found particularly challenging. Experienced adult researchers held regular meetings with youth researchers to review their data and to provide them with guidance and insights on improving data quality. Experienced adult researchers also collected data from parents and caregivers, health care workers and community members through 6 FGDs (n = 60 participants) and IDIs (n = 12). Qualitative discussions focused on among other things, challenges faced by HIV positive children, support systems for these children, disclosure, treatment and care, adherence plus future plans. The conduct of IDIs and FGDs involved an iterative process of data collection, on-going analysis and topic guide revision to test for theme saturation [43]. Data were also collected from the stakeholder meeting in the form of flip chart summaries of group discussions and note taking during plenary discussion and feedback.

### Data Analysis

Quantitative data were analysed using Stata 10 (Stata Corp., College Station, TX, USA). Adherence to ART was assessed through self-reports of frequency of missed doses (Table 1). Primary analysis included the “other” response as more adherent than those who reported missing their dose once per month, (responses were scaled in this way and the questionnaire did not provide a response “less than once per month”) (Table 1). A secondary analysis was performed, in which those participants responding “other” were excluded from the analysis.

**Table 1:** Demographic and adherence characteristics of study participants.

Variable	*n* (%)^1^
Gender	
Female	135 (59)
Median age (y)	14
Age range	6–19
Education	
Completed primary school (*n* = 167^2^)	111 (66)
ARV adherence Frequency of missed doses (% adherence) (*n* = 192^3^)	
Once a day (50%)	41 (21)
Twice a week (86%)	11 (6)
Once a week (93%)	11 (6)
Once every two weeks (96%)	7 (4)
Once a month (98%)	23 (12)
“Other”	99 (52)

1 *n* = 229 unless otherwise specified.

2 Of those 12 years of age and older.

3 Of those reporting that they were currently taking antiretrovirals.

To cross-tabulate adherence by other factors (independent variables, IVs) adherence was considered as a binary variable, where ‘adherent’ was defined as taking >95% of ARV doses i.e. missing no more than one dose every two weeks, and continuous IVs were also categorised. However, all testing for association was based on the ordered six categories of adherence and continuous values where appropriate for IVs such as age. Different tests were used to test for association between each IV and medication adherence: Wilcoxon Mann-Whitney test where the IVs were binary, Kruskal-Wallis test for categorical IVs with more than two levels, and Spearman’s rank correlation test for continuous or ordinal IVs. Ordinal logistic regression was used to calculate unadjusted odds ratios (OR) and corresponding 95% confidence intervals (CI). To examine the association between adherence and multiple IVs, multiple ordinal logistic regression was used to produce adjusted ORs and CIs. The two-sided *p*-value threshold for statistical significance was 0.05.

Common mental disorder (CMD) was assessed using a locally-validated, 14 item, indigenous measure of common mental disorders, the Shona Symptom Questionnaire (SSQ) [44]. A score of eight or higher suggested a risk of CMD whilst a score of 11 or higher suggested a risk of severe CMD [44].

Tape-recorded qualitative data were transcribed and translated verbatim into English. Initial themes were identified during the data collection process; these were used to develop an initial coding framework. Additionally, sets of five IDIs, FGDs and RoLs were coded line by line on paper. Additional codes were added to the coding framework. Transcripts were then entered into NVivo 8 (QSR International, Melbourne, Australia), a qualitative data storage and retrieval program. Two researchers (PC and MM) coded each transcription (and ‘river of life’) separately using the modified coding framework, taking note of any emerging new codes. Discrepancies were resolved by discussion with the senior social scientist (WM), who also independently coded all transcripts. Codes were grouped into categories and emerging themes were then identified following the principles of thematic analysis [45]. A total of 16 themes were identified. However, only those relevant to this analysis were used and illustrated with verbatim quotes.

Ethics approval was given by the Medical Research Council of Zimbabwe and the ethics board of University College London. Parental/guardian written consent was obtained for all participants. Additionally, written assent was required for adolescents aged 13 and older. Younger participants gave verbal assent; their names plus information on whether or not they had assented to take part in study were recorded. Researchers ensured that the wording of the consent/assent forms was appropriate for young people so that they became fully aware of the process. Researchers also strived to ensure that young people understood that they could refuse to participate in the study even when their parent/guardian had given consent. If any young person declined to assent to participation s/he would not be included in the study regardless of whether or not their parent/guardian had given consent. Participants were provided with copies of their informed consent/assent forms. Both ethics committees approved the consent/assent procedure. To ensure that participants had adequate support if they became upset or got disturbed by taking part in this study, an Africaid support group leader (experienced counsellor) was on hand at all interviews to provide further counselling. Additionally, a youth researcher support group was set up and led by an experienced counsellor and social scientists working on the project, to allow youth researchers to debrief and get support during data collection.

## Results

A total of 229 out of 310 (74%) young people enrolled in Africaid were located during the survey period, and all agreed to participate. All completed the quantitative questionnaire. No one declined to take part in the study. The median age was 14 years (interquartile range [IQR]: 12–16) and 135 (59%) were female (Table 1). The majority (75%) reported using their father’s family name; 88% had a birth certificate.

Orphanhood was common: 173 (76%) participants stated that their mother (21%), their father (38%) or both (40%) had died. The majority lived in households that were not headed by their mother or father (63%); only 65% had always lived in their current household. Among those who had changed households, 56% had moved once while 12 (15%) had moved more than three times, mostly due to caregiver’s illness or death (31%). Forty four respondents (19%) reported that they were unable to afford to eat at least two meals a day, nearly half (48%) that they would be unable to pay the required hospital fees if somebody in their household was sick, and two thirds that they had been absent from school because there was no money to pay school fees.

Eight participants reported never having attended school, with 184 (83%) in school at the time of questionnaire completion. Of 221 young people who had attended/were attending school, 50% had completed primary level to date (66% of those aged ≥12). The highest level of education reported was completion of 12 years of schooling (1%). The most commonly cited reason for stopping school was lack of fees (5/8). Qualitative discussions suggested that caregivers did not prioritise payment of fees for HIV positive children within their household. *‘They may deliberately not pay your schools fees such that you are always sent back home…’* (female, youth fgd1).

Participants perceived that they were treated differently from other children in their household. *‘…Whenever you get home [from school], you will find a whole pile of plates waiting for you and you will be asked to wash those plates, something which they won’t do to other children*’ (female, youth fgd3). Another youth mentioned, ‘*You can be given your own blanket – just one and so you will feel cold. Whenever you cough [due to cold], they tell you “shut up! You are making noise; we want to sleep”’* (male, youth fgd1).

Qualitative findings on household stigma were corroborated by quantitative data; 31% reported sometimes or always having had to work more (at home) than others their own age or younger, 34% had been asked, sometimes or always, to either sleep or stay separately from other household members and 41% were sometimes or always given less food than others their own age or younger.

Participants also perceived themselves stigmatised by the wider community. More than 40% of participants endorsed the statement that *“people do not want them around their children once their HIV status is known”*. Community discussions suggested that this discrimination was real *‘These young people are discriminated by caregivers, teachers and anyone within their vicinity’* (male, adult fgd1). Young people themselves described how other children from the community avoided them. *‘…If you try to get closer to them they will say, “don’t get close to us, you will infect us with AIDS”’* (female, youth fgd1).

Discussions suggested that while young people often felt that people avoided them at school, others had experienced teachers being supportive. *‘Some really feel sorry for you and if they find out that your classmates are abusing you, they can be very angry’* (male, youth fgd3). Overall, qualitative discussions highlighted the need to address stigma and discrimination within the wider community. Of note however, community members commented that the secrecy surrounding HIV positive children’s HIV status made it difficult for them (or indeed other household members) to provide support. *‘There are times when you want to help or even give advice but you will be afraid that they will ask “who told you that my child is positive (HIV)?”’* (female, community fgd2).

Knowing one’s HIV status is a requirement for participation in the Africaid support group. Nonetheless, only 90% of respondents reported knowing their HIV status. Participants reported that they were most commonly informed that they were HIV positive by their doctors (46%) or their mothers (22%). Perhaps not surprisingly given the extent of perceived and enacted stigma, 65% of the participants who knew their status had never told anyone else about it. The majority of discussants reported that they had chosen not to disclose their status, even to people who were important to them such as siblings and other household members, because they feared their reaction:

Interviewer: Why have you not told him (brother) that you are positive (HIV)?

Participant: He drinks and so my mother said we should not tell him because he might divulge the information to others when he gets drunk (male, youth IDI9).

Of note, adolescent girls also mentioned that they rarely chose to disclose to the boys they dated. ‘You cannot disclose to someone you have known for just a week. He will disappear for good and he will tell his friends that he is no longer seeing you because you are HIV positive’ (female, youth fgd2).

Qualitative data suggested that apart from parental illness and death, becoming aware of their HIV status is perhaps the most difficult life event the young people have experienced. All participants who completed the ‘river of life’ represented this period with a deep plunge on the river. Young people reported that learning their HIV status resulted in feelings of despair and hopelessness, coupled with a sense of imminent death. One participant described how he temporarily gave up. ‘After learning about my status I spent a week without going to school. I lost appetite…’ (male, youth IDI10).

Eighty-nine (63%) of the 141 participants (aged 13 or over) who completed the SSQ scored eight or higher - suggesting that they were at risk of common mental disorder (CMD) [44]. Forty-two (30%) scored 11 or more, suggesting a risk of severe CMD. The median score was 9 (mean 8.2, SD 3.7). Items in the scale most likely associated with symptoms of depression were commonly reported; e.g. 72% agreed that ‘in the past week there were times when I was thinking deeply or thinking about many things’ (kufungisisa [thinking too much] is the Shona word that most closely resembles the English term ‘depression’) (Table 2). Qualitatively, participants mentioned that the ill-treatment they experienced within the household made them ‘think too much’. ‘You start saying to yourself, “I wish my mother had twisted my neck when she was about to die so that both of us would die at the same time”’ (female, youth fgd1).

**Table 2:** Shona Symptom Questionnaire.

Items	Yes (%) (n = 141)^1^
There were times in which I was thinking deeply or thinking about many things	102 (72)
I found myself sometimes failing to concentrate	93 (66)
I lost my temper or got annoyed over trivial matters	85 (60)
I had nightmares or bad dreams	98 (70)
I sometimes saw or heard things which others could not see or hear	46 (33)
My stomach was aching	100 (71)
I was frightened by trivial things	72 (51)
I sometimes failed to sleep or lost sleep	88 (62)
There were moments when I felt life was so tough that I cried or wanted to cry	107 (76)
I felt run down (tired)	105 (74)
At times I felt like committing suicide	37 (26)
I was generally unhappy with things that I would be doing each day	79 (56)
My work was lagging behind	66 (47)
I felt I had problems in deciding what to do	76 (54)
Median SSQ	9
Number scoring ≥8 (risk of common mental disorder)	89 (63)
Number scoring ≥11 (risk of severe common mental disorder)	42 (30)

1 Represents all participants 13 years of age or older, and includes four 12 year olds who were mistakenly administered ACASI instead of CAPI as indicated (as discussed in notes for *Fig. 1*).

Twenty-one percent of participants reported missing a dose of their ART once a day (50% adherence), 6% missed twice a week (86% adherence), 6% missed once a week (93% adherence), 4% once every two weeks (96% adherence) and 12% once a month (98% adherence). Ninety-nine participants (52%) selected ‘other’ which was the last option in a list that progressed from least to greatest adherence, and did not include an option labelled ‘less than once a month’ to indicate near-perfect adherence. Qualitative discussions suggested that participants who had chosen this option intended to indicate near perfect adherence. Overall, 16% of the sample reported that they took over 95% of their doses (the recommended adherence level) [46] (Table 1).

Among 192 participants, the most common reasons for missing doses were forgetting (45%), travelling (23%), concealing from others (22%) and lack of bus fare to collect medication (17%). Older age and psychological morbidity appeared to be associated with poor adherence. Whilst 76% of participants aged under 13 reported taking >95% of ART doses, this declined to 55% in those aged 16 and over. Similarly, 72% of participants who scored <8 on the SSQ (low risk of CMD) reported good adherence declining to 53% of those whose who scored 11 or more (Table 3). In a multiple ordinal logistic regression model neither age (adjusted odds ratio per year increase 0.92, 95% CI 0.76–1.11) nor SSQ score (adjusted odds ratio per unit increase 0.92, 95% CI 0.84–1.00) was found a significant predictor of medication adherence after adjusting for the other.

**Table 3:** Associations with adherence.

Factor	Adherent,n (%)	*p*-value	Unadjusted OR^1^(95% CI)
*Total*	129 (67)		
Gender			
Female	77 (69)	0.534	1.48 (0.87–2.53)
Male	52 (64)		1 -
Age, years			
<13	51 (76)	<0.001	0.86 (0.77–0.95)^2^
13–15	48 (69)		
>16	30 (55)		
Education			
Completed primary school	64 (64)	0.438	0.90 (0.44–1.86)
Did not complete primary school	60 (70)		1 -
Sufficient essential items			
Some lack of items	105 (68)	0.567	1.00 (0.50–1.98)
No lack of items	24 (63)		1 -
Food security			
Some insecurity	60 (66)	0.760	0.78 (0.46–1.34)
No insecurity	69 (68)		1 -
Orphanhood			
Not orphan	29 (63)		1 -
Single^3^	60 (68)	0.794	1.09 (0.56–2.12)
Maternal^3^	63 (68)	0.475	1.27 (0.66–2.46)
Paternal^3^	77 (69)	0.576	1.20(0.63–2.27)
Double^3^	40 (69)	0.416	1.35 (0.65–2.81)
Changes to household			
Changed at least once	52 (75)	0.080	1.69 (0.96–2.96)
No change	77 (63)		1 -
Sickness in family			
Sick family members	58 (67)	0.874	1 -
No sick family members	58 (64)		0.91 (0.52–1.58)
Disclosure			
Disclosure	38 (61)	0.242	0.70 (0.40–1.25)
No disclosure	78 (70)		1 -
Mental Health (SSQ)			
<8	34 (72)	0.038	0.92 (0.84–1.00)^2^
8–10	23 (56)		
≥11	19 (53)		
Stigma			
None	23 (74)	0.855	1.05 (0.87–1.27)^2^
1–5	65 (68)		
All	5 (71)		
Transport fares to collect ARVs			
Lack of fares	22 (69)	1.000	1 -
No lack of fares	107 (67)		0.84 (0.42–1.67)

1 ORs for increasing adherence as measured by a scale, calculated under the assumption of proportional odds.

2 OR for a one unit increase in factor.

3 Orphanhood categories not mutually exclusive. ORs and p-values for each category calculated relative to non-orphans.

Discussions with adults suggested that poor adherence among adolescents was sometimes due to a lack of monitoring. ‘I cannot follow-up a 14 year old to check whether or not they are taking their tablets’ (female, adult fgd6). However, adults also mentioned that they felt ill-equipped to support adolescents with adherence. Additionally, FGDs also suggested that non-adherence was sometimes a conscious act, triggered by depression. ‘You end up thinking too much and at times you say, “Let me stop taking these tablets so that I just die”’ (male, youth fgd3).

A majority of participants attended Africaid support group meetings at least once every month (95%). Nearly all participants (91%) found being a member of the support group helpful. Qualitative discussions suggested that being a support group member gave young people a sense of belonging. ‘*When I went to the support group, I learnt that there were other young people like me and when they shared their experiences, I felt much better and got the comfort that after all, I was not alone*’ (male, RoL13). However, young people stressed that life outside the confines of the support group was more challenging. For example, there was a lack of understanding of these young people’s issues at home characterised by ignorance about ARVs, safety of dating and possibility of future aspirations.

### Stakeholder Discussion

The meeting to discuss and verify results was attended by the research team, adolescents with HIV, Africaid staff, experienced programmers including health care providers, UNICEF and officials from the Zimbabwean Ministries of Social Welfare and Health. The results were presented and their interpretation was discussed, first in groups and then in plenary. Stakeholders felt that the data suggested that psychosocial support for HIV positive adolescents needed to be expanded from its focus on supporting individual adolescents to a more family/household centred approach in order to address some of these broader contextual issues. They recommended a multi-component approach that combined increased access to individualised support to address the mental health and adherence needs of adolescents with an intervention that actively involved caregivers in order to strengthen their knowledge and skills.

### Enhancing the Existing Africaid Intervention

**Figure 2:**
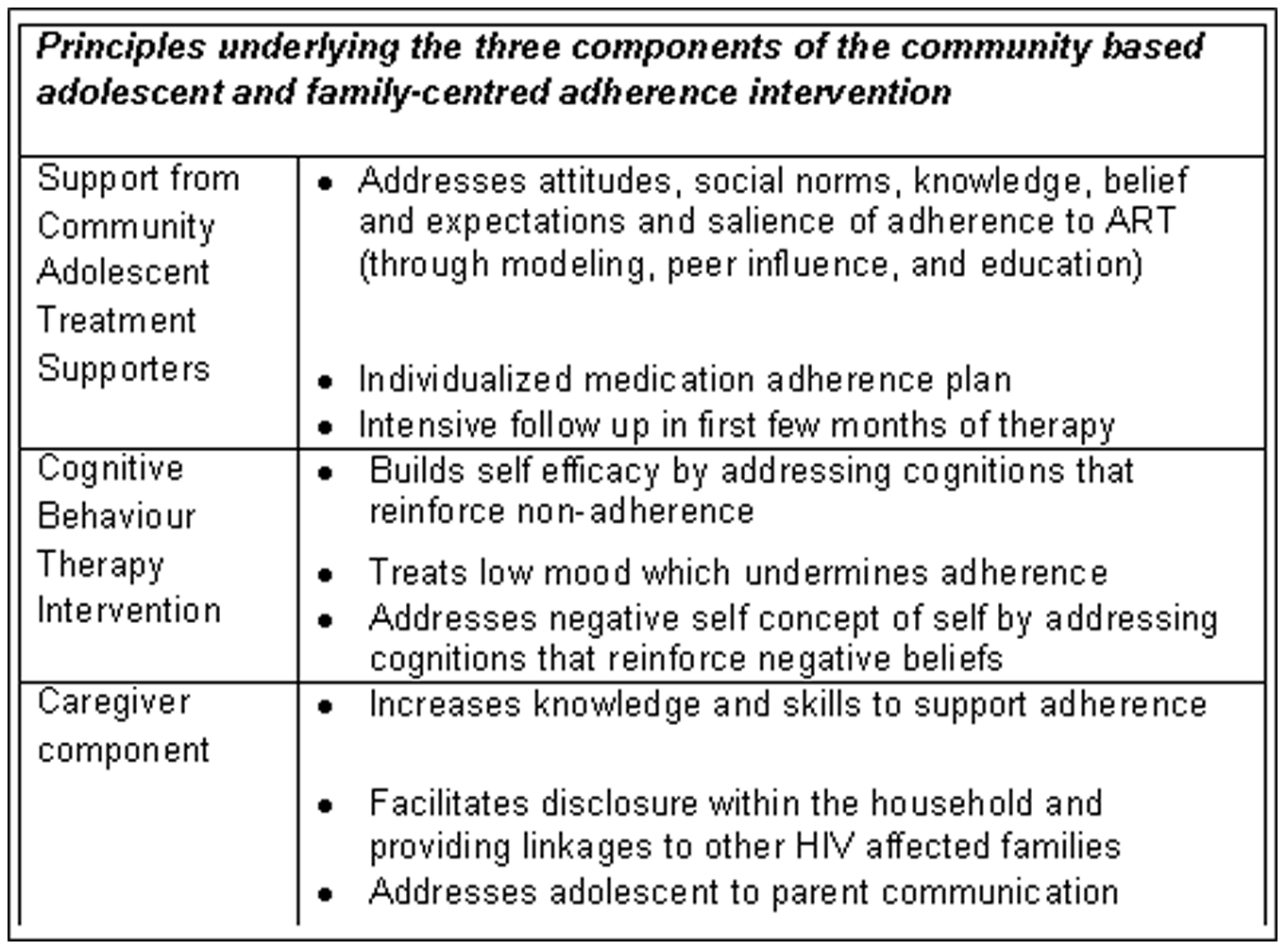
Three components of the community based intervention.

Based on the stakeholder discussions we identified a validated conceptual framework, the Unified Theory of Behavior [34], [35], that had previously been adapted to support adolescent behavior change within the context of a family centered approach. The UTB conceptualizes behaviour in terms of two dimensions; firstly the immediate determinants of behaviour and secondly the immediate determinants of behavioural intention. It states that adherence to ART (for example) is more likely to occur if both i) the determinants of adhering to ART and ii) the intention to adhere to ART are ‘aligned in favour of its enactment’ [34], [35]. We adapted this framework by combining evidence from the literature about the determinants of adolescent HIV adherence with the data from our formative work to guide expansion of the intervention (Figure 2). We endeavoured to ensure that each component of the framework was adequately addressed in the expanded programme.

## Discussion

This mixed methods research was useful in highlighting the dominant issues in the lives of HIV positive young people. While the survey data allowed us to explore and quantify a number of important issues, qualitative data allowed us to obtain a more nuanced understanding of the reality of these young peoples’ lives, highlighting the value of combining the two methods in order to obtain more in-depth, balanced findings [47], [48], [49], [50].

This is one of the first studies from a country with a generalised epidemic to report life circumstances of HIV positive young people. It tells a story of internal and external challenges these young people face. Given the challenges they report, it is not surprising that a high proportion of those screened were at risk of common mental disorder and in particular, depression. Poor psychological health, as well as being an important issue in its own right, is strongly associated with other health and social problems including low educational attainment, unemployment, alcohol/drug abuse, risky sexual behaviours, sexual abuse and poor sexual/reproductive health [51], [52], [53]. In the context of HIV, young people with poor mental health face additional challenges in relation to medication adherence and to the consequences of increased sexual risk-taking [54], [55]. In adults, there is increasing recognition of the importance of mental health in relation to HIV transmission [55], [56]. However, to date few HIV prevention interventions have included a mental health component and even fewer of these have been conducted in adolescents.

Our study clearly shows that even self-reported medication adherence in this group is sub-optimal. Taking medication for any chronic disease is challenging and, for non-HIV related chronic childhood illnesses (e.g. diabetes, asthma), compliance with drugs frequently becomes worse as the child becomes more independent from parental control as they enter adolescence [25]. Research demonstrated that adolescents in southern Africa have poorer ART adherence than adults [26], [27]. In a large American cohort of adolescents, factors associated with non-adherence were increasing age, female gender, detectable HIV viral load, recent stressful life events, and diagnosis of mental health disorder such as depression or anxiety [57].

A systematic review of 17 studies relating to ART programs in children and adolescents from low and middle income countries found median adherence ranging from 49–100% [58]. Factors associated with level of adherence were investigated in eight studies. Associations were found between medication adherence and the child’s socioeconomic environment, stigma, caregiver characteristics, dynamics of child-caregiver interactions, disclosure of HIV status, regimen complexity, side effects, and cost of medication [58]. The recently updated US Paediatric HIV Guidelines include recommendations to improve medication adherence in adolescents including use of individualized treatment plans, intensive follow-up in the first few months of therapy, and simplifying drug regimens among other things [46].

In the Zimbabwean context, our data confirmed the association between poor adherence and the wider psychosocial environment in which the adolescent lives and pointed to the need for a family/household centred approach to providing support which addresses both individual issues relating to the logistics of adhering to ART and the wider contextual barriers such as disclosure, stigma and depression.

The Unified Theory of Behaviour (UTB) has been previously validated as a framework to underpin family-centred interventions for adolescents [34] in order to support behaviour change. We adapted the UTB to adolescent adherence to HIV treatment and care in Zimbabwe to develop a culturally-relevant adolescent and family-centred intervention for promoting ART adherence (Figure 2). The expanded intervention combines three components i) a monthly community based support group with individualized support from a Community Adolescent Treatment Supporter (CATS); ii) a caregiver intervention; iii) a group cognitive behavioural therapy intervention to support adherence. These multiple components address both the determinants of the intention to adhere (which mainly relate to psychosocial aspects) as well as of adherence per se (related to knowledge, skills and environment) and are intended to be overlapping and synergistic.

The monthly community-based support groups will led by a ‘volunteer’ nurse counsellor with individualized support provided through Community Adolescent Treatment Supporters (CATS). These CATS are older adolescents with HIV who are trained and supported (using MoHCW guidelines for lay counsellors) to provide support for both adherence and more general issues facing HIV positive adolescents (such as information about HIV generally, issues related to disclosure, grief, stigma, dating and their sexual and reproductive health). This approach combines the principles of 'positive health dignity and prevention' [59] with the characteristics of effective adherence interventions outlined in a recent Cochrane review: practical medical management, individualized support, intensive follow-up in the first few months of therapy and intervention of more than 12 weeks duration [60]. b) In addition, we have adapted and tested a 10 session cognitive behavioural therapy (CBT) intervention to improve self-efficacy and emotional resilience among Zimbabwean teenagers, [61], [62], [63] as well as specifically address negative cognitions and beliefs, which is being further adapted to include material related to adherence to ART [64].

This CBT intervention is the only ‘preventive mental health program’ recommended by WHO for use in adolescents [65] and CBT is one of the few psychological approaches that has been successfully used to improve ART adherence among adults with depression. c) Importantly, the program will also address support for caregivers and other household members. Our data show that it is essential to involve the wider family unit in the care of these young people both to try and reduce stigma and discrimination in the household and through that, the wider community, and to enhance support for all aspects of their treatment and care. Of note, as advocated by Reisner and colleagues [66], this approach specifically takes account of the wider contextual issues facing young people and does not ‘just focus’ on adherence.

This evidence-based, theoretically-derived adolescent and family-centred adherence support program is sustainable and affordable locally (and indeed is currently being scaled up within Zimbabwe through Africaid). It is a potentially portable intervention that could be used across sub-Saharan Africa. We plan to evaluate the effectiveness and cost-effectiveness of this theoretically-derived approach in terms of its impact on psychological well-being, HIV outcomes, ART adherence and reported sexual risk-taking as the programme is expanded.

Separately there is growing recognition of the need to involve youth researchers in research design, conduct and analysis [67], [68]. The general belief is that this will likely enable greater equity in the research process, eliminating the hierarchical power dynamics which characterize much research with young people, and which can characterize research conducted by ‘Northern’-led teams in resource-limited settings [67], [69]. Although youth researchers were able to collect quantitative data, their interviewing skills were sub-optimal (e.g. use of probes was generally poor). Some of the data had to be recollected by more experienced researchers. Participatory tools, such as the ‘river of life’, which we used to obtain life history narratives, appeared to be more intuitive and resulted in higher quality data collection. Youth researchers had valuable insights that helped in data interpretation. Overall, we found that whilst there are benefits in engaging youth researchers, the process is not without its challenges and needs to be complemented by other data collection approaches.

In conclusion, there is clearly an urgent need to develop and carefully evaluate culturally-appropriate and sustainable strategies to improve and maintain child and adolescent adherence plus psycho-social support for this vulnerable group. This mixed methods research allowed us to describe the lived experiences of HIV positive young people in Zimbabwe which were then used to inform the adaptation of Africaid’s intervention to develop a more theoretically-derived and comprehensive programme of support which we plan to evaluate in terms of its efficacy and cost-effectiveness as it is brought to scale.
